# Intrinsic Valuation of Information in Decision Making under Uncertainty

**DOI:** 10.1371/journal.pcbi.1005020

**Published:** 2016-07-14

**Authors:** Daniel Bennett, Stefan Bode, Maja Brydevall, Hayley Warren, Carsten Murawski

**Affiliations:** 1 Melbourne School of Psychological Sciences, The University of Melbourne, Parkville, Victoria, Australia; 2 Department of Finance, The University of Melbourne, Parkville, Victoria, Australia; Oxford University, UNITED KINGDOM

## Abstract

In a dynamic world, an accurate model of the environment is vital for survival, and agents ought regularly to seek out new information with which to update their world models. This aspect of behaviour is not captured well by classical theories of decision making, and the cognitive mechanisms of information seeking are poorly understood. In particular, it is not known whether information is valued only for its instrumental use, or whether humans also assign it a non-instrumental intrinsic value. To address this question, the present study assessed preference for non-instrumental information among 80 healthy participants in two experiments. Participants performed a novel information preference task in which they could choose to pay a monetary cost to receive advance information about the outcome of a monetary lottery. Importantly, acquiring information did not alter lottery outcome probabilities. We found that participants were willing to incur considerable monetary costs to acquire payoff-irrelevant information about the lottery outcome. This behaviour was well explained by a computational cognitive model in which information preference resulted from aversion to temporally prolonged uncertainty. These results strongly suggest that humans assign an intrinsic value to information in a manner inconsistent with normative accounts of decision making under uncertainty. This intrinsic value may be associated with adaptive behaviour in real-world environments by producing a bias towards exploratory and information-seeking behaviour.

## Author Summary

Acquiring information about the external world is vital for planning and decision making. However, recent research has shown that some animals choose to acquire information at considerable cost even when the information is of no practical benefit, a counter-intuitive behavior associated with suboptimal outcomes. In this study, we demonstrate that humans also engage in this suboptimal behavior by forfeiting future monetary reward in exchange for early but unusable information about future outcomes. Our results suggest that participants attach a value to information beyond its purely instrumental value. This preference for information may help account for apparent anomalies in human choice behavior such as compulsive checking behaviors in obsessive-compulsive disorder, and excessive and wasteful use of uninformative laboratory testing in hospitals.

## Introduction

In many decision situations, agents possess only incomplete information about decision outcomes, and may choose to seek out further information before choosing a course of action [1, 2]. For instance, a surgeon considering whether to operate on a tumor might first request a biopsy to determine whether the tumor is malignant or benign. Despite being a key feature of choice problems in natural settings, information seeking is not considered within many standard accounts of decision making under risk and uncertainty [3–5]. Moreover, it has been shown that some animals choose to seek information even when that information cannot be used to improve future outcomes [6–8]. This behaviour, which is suboptimal from the perspective of expected reward maximization, suggests that biological agents may attach a value to information which is not solely defined in terms of tangible future outcomes [9].

Historically, many theories of information valuation have adopted an *instrumental* framework, in which the value of information is calculated solely in terms of expected instrumental benefit [10–12]. These theories predict that a decision-maker should seek information only if the information is expected to impart a tangible benefit in excess of its cost [11]. For instance, a clairvoyant charging $100 to reveal whether stock prices will rise or fall should only be consulted if a payoff greater than $100 is expected to result from using this information. Instrumental valuation of information is normatively optimal, in the sense that it maximises expected monetary reward. However, one strong prediction of instrumental valuation is that information of no instrumental use for acquiring payoffs (henceforth termed *non-instrumental information*) should not affect choice behaviour. As a result, instrumental valuation of information cannot easily explain curiosity-driven or purely exploratory behaviours [13, 14].

An alternative proposal is that biological agents may attach an *intrinsic* value to information, such that information about relevant future outcomes is valued for its own sake, independent of direct, tangible payoffs [15]. Similarly, economic decision theory has posited that humans might possess a preference for early resolution of uncertainty which would result in intrinsic value of information [16–18], and recent theories of active inference propose that choice behaviour can be explained by sensitivity to information gain as well as to extrinsic reward [19]. In support of intrinsic valuation of information, human participants have been shown to prefer early to late information about receiving an unavoidable electric shock [20], and to be conditioned by non-instrumental information in a behavioural conditioning paradigm [21]. Moreover, neural data from humans and non-human primates have shown that non-instrumental information is encoded using similar mechanisms, and within similar circuits, to primary and monetary reward [9, 22–24]. These findings are consistent with the hypothesis that biological agents assign an intrinsic reward value to non-instrumental information about future outcomes using a coding scheme commensurate with primary and monetary reward.

One limitation of previous empirical work assessing preference for information in humans is that information available in decision-making tasks is usually of instrumental benefit to participants, such that it is difficult to dissociate the intrinsic value of information from its instrumental value [25, 26]. To address this issue, the present study adapted a task from the animal literature which allowed preferences for non-instrumental information to be elicited in a well-controlled and incentive-compatible manner [22]. Using this task, we sought to test one counterintuitive prediction of intrinsic valuation of information: that, like starlings and pigeons, human participants would trade off information against extrinsic reward by sacrificing part of an uncertain future reward in exchange for early but non-instrumental information about reward likelihood [27].

Furthermore, among theories positing an intrinsic value of information, the source of this value is often unspecified. For instance, the Kreps-Porteus model in economic decision theory predicts a preference for early resolution of uncertainty from a particular axiomatic formulation of utility, but does not specify a cognitive mechanism which might drive this preference [16]. One proposal is that preference for non-instrumental information might result from an aversion to temporally prolonged uncertainty, such that agents may seek information in order to obtain relief from uncertainty [13, 28–30]. We therefore tested a novel computational cognitive model, which assumed that inter-individual variability in the intrinsic value of information resulted from stable trait-level individual differences in degree of aversion to uncertainty, against a standard expected reward maximization model, which assumed that information was assigned solely instrumental value [11]. Finally, in order to determine whether the duration of uncertainty affected participants’ preference for information, we also conducted an additional experiment in the rate at which non-instrumental information was delivered was experimentally manipulated.

## Results

**Fig 1:**
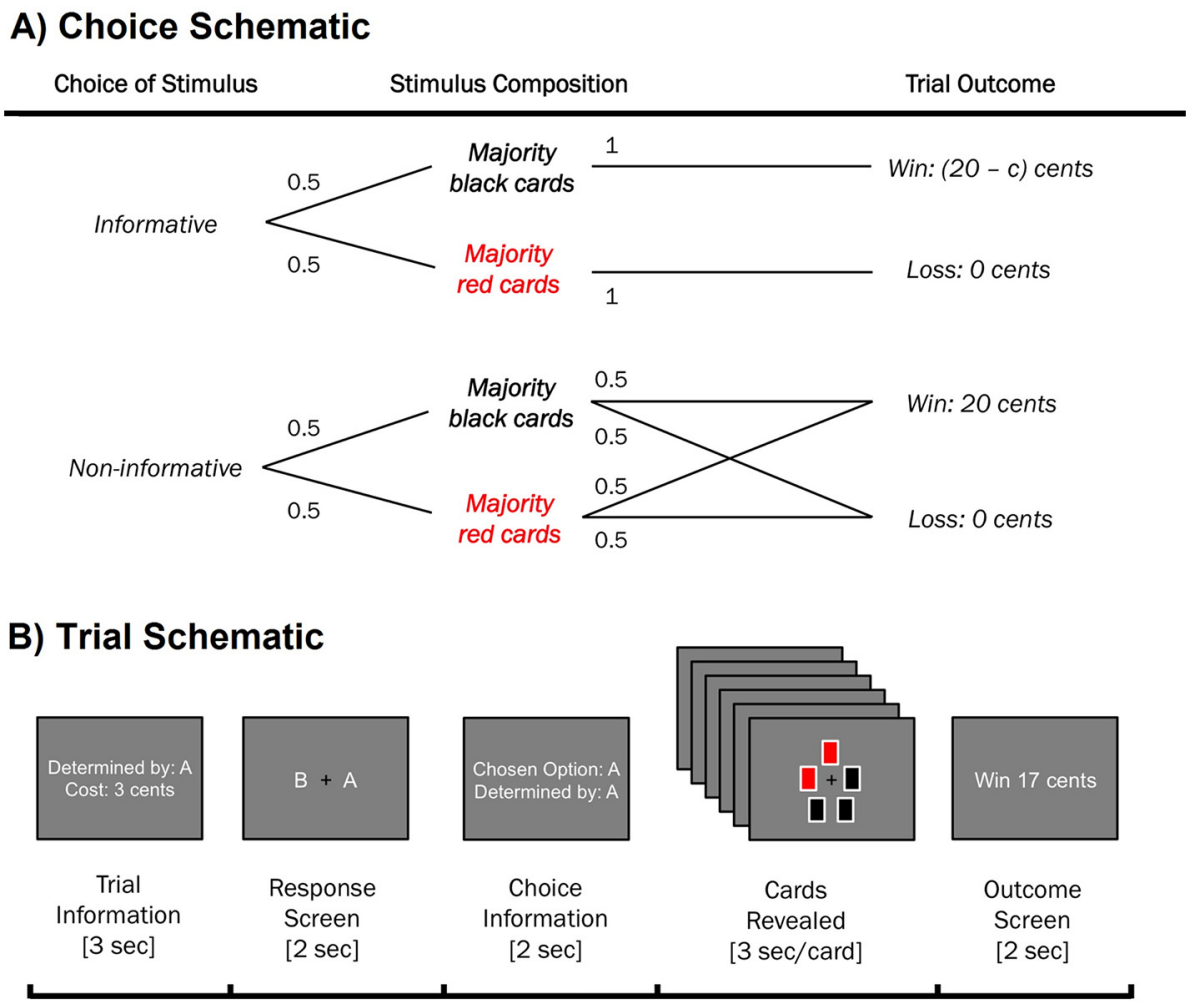
Task schematic. (A) Choice schematic. In the informative stimulus, the colour composition of cards perfectly predicted lottery outcome. In the non-informative stimulus, cards had no predictive validity for the lottery outcome. The information cost *c* was subtracted from lottery winnings only in the case of a win outcome. As a result, a loss always resulted in the same outcome (receive 0 cents) regardless of the participant’s choice of stimulus. (B) Trial schematic. Participants first received information regarding the identity and cost of the informative stimulus (both counterbalanced across trials), and then made a choice using left and right arrow keys within 2 seconds (left/right mapping of A and B counterbalanced across trials). Participants were then presented for 2 seconds with a choice information screen, following which cards from the chosen stimulus were revealed sequentially at a constant rate of 3 seconds per card (18 seconds total delay). Participants were informed that all outcomes were predetermined, and that choice of stimulus was unrelated to win probability. If the participant failed to respond during the choice window, the non-informative stimulus was shown and no reward was subsequently delivered.

To titrate preferences for non-instrumental information in human participants, we developed a novel variant of an experimental task used in animal research [7, 8, 22]. In each trial of this task, a lottery was played out in which participants could either win (receiving 20 cents) or lose (receiving 0 cents), with equal probability (see Fig 1a). Participants were asked to express their preference for observing one of two stimuli (termed ‘Set A’ and ‘Set B’) in the delay period prior to the presentation of the lottery outcome. Both stimuli took the form of five-slot arrays of red and black cards, with card colours initially hidden and then revealed one-by-one at a constant rate (see Fig 1b). One of the two stimuli (the ‘informative stimulus’) imparted information regarding the lottery outcome: a majority of black cards indicated that the participant would win, whereas a majority of red cards indicated a loss. By contrast, the other stimulus (the ‘non-informative stimulus’) consisted of five black and red cards whose colours were determined pseudo-randomly, and which therefore imparted no information about the lottery outcome. Informative and non-informative stimuli were therefore perceptually equivalent, but only the informative stimulus imparted information regarding the lottery outcome. Crucially, the information gained by observing the informative stimulus was non-instrumental, since it affected only the participant’s certainty regarding the lottery outcome, not the probabilities of the lottery itself.

To assess participants’ willingness to sacrifice monetary reward for non-instrumental information, on each trial a monetary cost was associated with the informative stimulus. Four cost conditions were assessed: 0 cents (free information), 1 cent, 3 cents, and 5 cents. If participants chose the informative stimulus, this cost was deducted from lottery winnings in the case of a win outcome, but not in the case of a loss outcome (see Fig 1a). Participants could not lose money by observing the informative stimulus, ensuring that information preference was not confounded by loss aversion. Since lottery probabilities were unaffected by participants’ choice of stimulus, expected reward was greater for the non-informative stimulus than the informative stimulus in all non-zero cost conditions. In the zero-cost condition, the expected monetary reward of informative and non-informative stimuli was equal.

We conducted two separate experiments using this paradigm. In Experiment 1, only information cost varied between trials. In Experiment 2, both information cost and information rate (the speed at which cards were revealed) differed between trials.

### Preference for non-instrumental information

**Fig 2:**
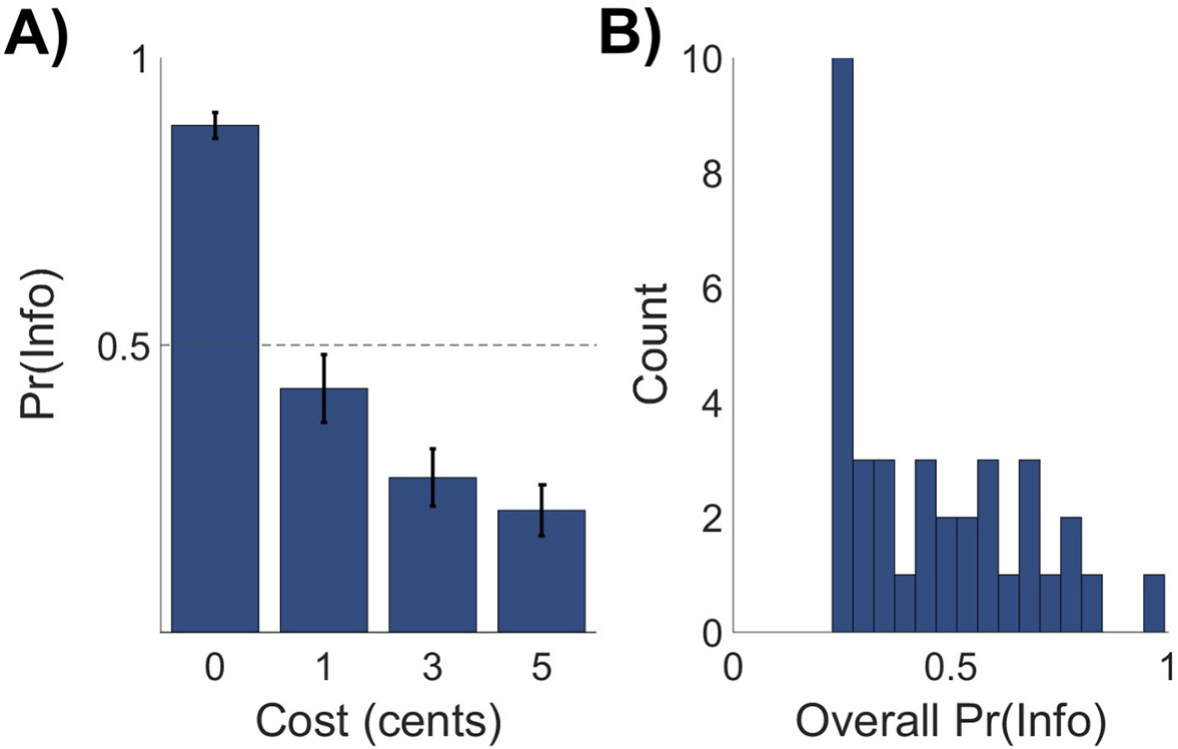
Behavioral results for Experiment 1. (A) Mean proportion of informative stimulus choices (denoted *Pr(Info)*) as a function of information cost. Error bars represent the standard error of the mean (SEM). Mean proportion of information-seeking choices was monotonically decreasing in cost of information. (B) Histogram of overall proportion of informative stimulus choices across participants, demonstrating inter-individual differences in behaviour. N = 40.

Experiment 1 assessed participants’ willingness to forfeit monetary reward in exchange for non-instrumental information, and examined the effect of information cost on information preference. Across cost conditions, participants chose the informative stimulus on 43.95 percent of trials (*SD* = 20.28), while showing good task engagement as evidenced by a low proportion of missed responses (*M* = 1.67 percent, *SD* = 1.72). On average, across cost conditions participants sacrificed 2.87 percent of available winnings in exchange for early information about the lottery outcome (*SD* = 3.21). A one-way repeated-measures analysis of variance (ANOVA) revealed that choice proportions were modulated by the cost of information (*F*(1.89, 73.60) = 65.68, *p* < .001; partial *η*^2^ = 0.63; see Fig 2a), with information choice proportion monotonically decreasing with increases in information cost. Control analyses revealed that behaviour was not significantly affected by the key used to select responses (left versus right arrow: *t*(39) = -0.71, *p* = .24) or the nominal identity of the informative stimulus (A versus B: *t*(39) = 1.40, *p* = .08).

We next used post-hoc *t*-tests with Bonferroni correction to assess whether participants’ behavior was consistent with expected reward maximization. Expected reward maximization, which implies solely instrumental valuation of information, predicts that participants should be indifferent between the informative and non-informative stimulus when information is free, and that the non-informative stimulus should dominate the informative stimulus for any non-zero information cost [11]. In the zero-cost condition, informative stimulus choice proportion was significantly greater than the indifference point of 0.5 (*t*(39) = 16.83, *p* < .001). In each of the non-zero cost conditions, informative stimulus choice proportion was significantly greater than zero (1-cent condition: *t*(39) = 7.17, *p* < .001; 3-cent: *t*(39) = 5.41, *p* < .001; 5-cent: *t*(39) = 4.76, *p* < .001). These results indicate that participants sacrificed future reward for early information, which is inconsistent with expected reward maximization. In addition, we observed notable individual differences in patterns of information seeking behaviour (see Fig 2b), indicating heterogeneity of task strategies between participants.

### Computational model of intrinsic value of information

To formalise the comparison between instrumental and intrinsic theories of information valuation, we implemented these theories as competing computational cognitive models, and assessed which model provided the best account of both group- and individual-level data.

**Fig 3:**
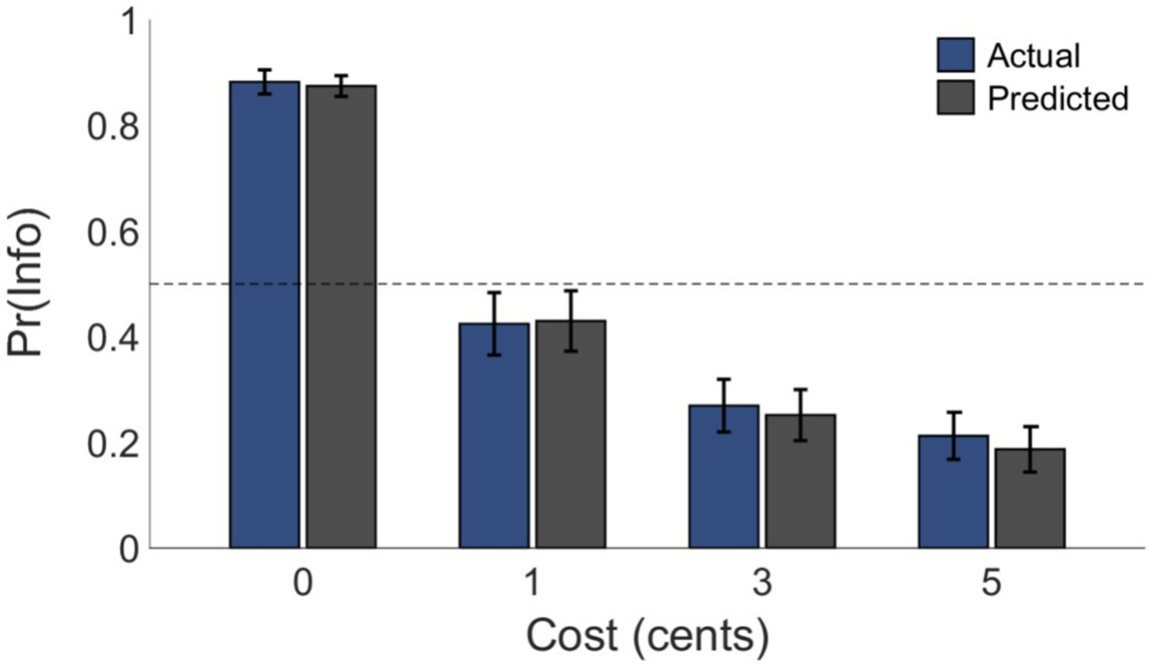
Model fit. Actual (blue) and UP-predicted (grey) group-level mean proportions of information-seeking choices as a function of information cost across participants. Error bars represent SEM. N = 40.

**Fig 4:**
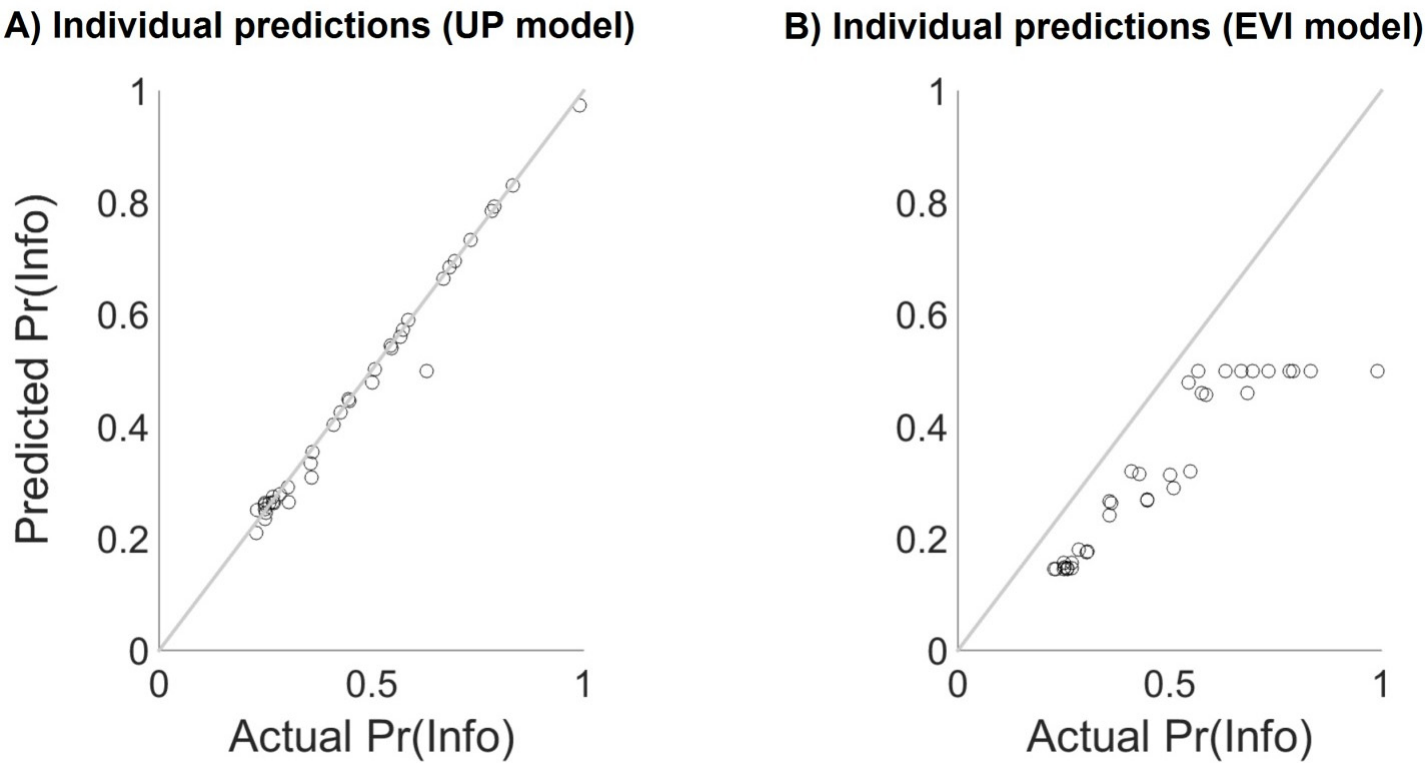
Individual-participant model fits. (A) Actual informative choice proportion, denoted Pr(Info) (horizontal axis) versus informative choice proportion as predicted by the UP model (vertical axis). Each circle indicates one participant. Euclidean distance from the diagonal (grey line) represents error in prediction. (B) Actual informative choice proportion (horizontal axis) versus informative choice proportion as predicted by the EVI model (vertical axis). Each circle indicates one participant. Euclidean distance from the diagonal (grey line) represents error in prediction. Across all participants, the EVI systematically under-predicted informative choice proportions (all participants fell below the diagonal).

The two models we assessed were termed the Expected Value of Information (EVI) model, which incorporated solely instrumental valuations of information, and the Uncertainty Penalty (UP) model, which also incorporated intrinsic valuation of information by assuming that the source of information’s intrinsic value was an aversion to temporally prolonged uncertainty [13, 28–30]. Both models considered the task in a Markov Decision Process (MDP) framework, and differed only in choice of state value function [31] (see also *Experimental Protocols*). We found that, in addition to providing the best overall account of choices across participants (smallest overall Bayesian Information Criterion (BIC) value; see Table 1 and Fig 3), the UP model provided the best fit for a large majority of individual participants. Accordingly, a likelihood-ratio test revealed that including the participant-specific uncertainty penalty parameter *k* greatly improved the overall fit of the UP model relative to the EVI model (χ^2^(40) = 1338.34, *p* < .001). Moreover, the UP model provided an unbiased fit to the data of all participants, including those who displayed a relatively weak overall preference for information (see Fig 4*)*. By contrast, the EVI model systematically underestimated informative stimulus choice proportions across all participants.

**Table 1:** Behavioural model fits for 4405 choices by 40 participants.

Model	Free parameters (per participant)	*-LL*	*BIC*	McFadden’s R^2^	*n* best fit
EVI	1	934.06	2212.13	0.51	3
UP	2	264.89	1209.40	0.86	37

*-LL*: negative log-likelihood. *BIC*: Bayesian Information Criterion. Numbers in the *n* best fit column are based on a comparison of individual-participant BIC values for each model.

Furthermore, we found that the best-fitting values of the UP’s scaling parameter *k* were greater than zero across participants (Wilcoxon signed-rank test: *Z* = 5.51, *p* < .001) and, in addition, were strongly correlated with overall proportion of information-seeking choices across participants (Spearman’s rho = 0.95, *p* < .001). This indicates that participants with a stronger aversion to uncertainty (higher *k* values) assigned a greater intrinsic value to information, and therefore made more information-seeking choices (see also *SI* section 1 for individual parameter estimates and parameter-behaviour correlations). Although unsurprising given the structural design of the UP model, the strength of this relationship serves to demonstrate that the UP model parameter designed to capture individual differences in information preference succeeded in doing so.

**Fig 5:**
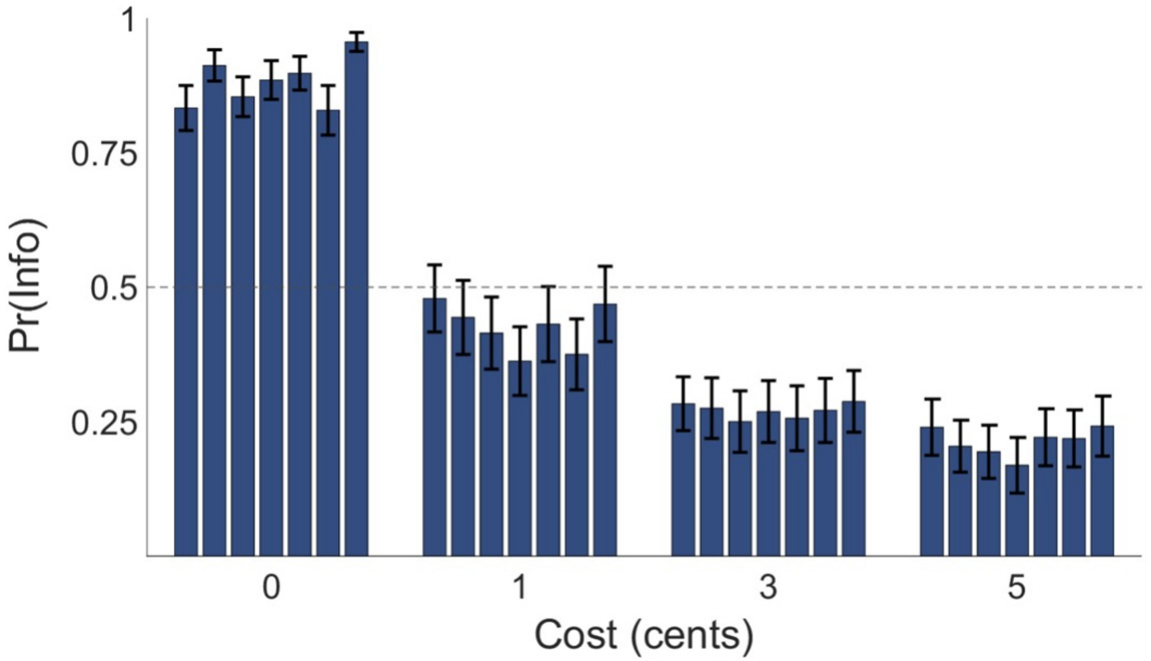
Block-wise behavioural results for Experiment 1. Mean proportion of information-seeking choices, denoted *Pr(Info)*, as a function of information cost and block number across participants. Choice proportions for blocks one to seven are presented in ascending order left to right within each of the four cost conditions. Error bars represent SEM. Preference for information was static across the task.

In addition, since the UP model’s implementation of intrinsic valuation of information assumes that aversion to uncertainty is a stable trait of participants, a secondary prediction of this model is that information’s intrinsic value ought to be stable across time for each participant. In order to test this prediction, we calculated information choice proportion separately in each of the seven experimental blocks in Experiment 1, and assessed the effect of block number on information preference using a 4×7 repeated-measures ANOVA with within-subjects factors of information cost (0, 1, 3, 5 cents) and task block (1 to 7). We found no significant main effect of task block on information choice proportion (F(3, 117.37) = 1.73, *p* = .16) and no significant interaction between task block and information cost (*F*(9.58, 373.59) = 1.05, *p* = .40). These results indicate that informative stimulus choice proportions did not differ significantly across the task (see Fig 5), as predicted by the UP model.

Finally, we performed an additional control analysis to ensure that the relative advantage of the UP model relative to the EVI model was not simply due to its better performance in the zero-cost condition. To this end, we repeated the model-fitting procedure while excluding all trials in the zero-cost condition (that is, the models were fit solely on the basis of the 1, 3, and 5-cent cost conditions). Once again, we found that the UP model (BIC = 718.31) provided a significantly better fit to data than the EVI model (BIC = 1031.60; likelihood ratio test: χ^2^(40) = 489.88, *p* < .001). This indicates that the UP model, which incorporated intrinsic valuation of information, outperformed the EVI model even when zero-cost trials were excluded from analysis.

### The effect of information rate on preference for information

A logical consequence of intrinsic valuation of information is that the temporal profile of uncertainty ought to affect participants’ preference for observing an informative stimulus. In particular, the same amount of information should have a different value to participants depending on the rate at which it resolves uncertainty. In Experiment 2, we tested this prediction among a new sample of participants. In Experiment 2, cards could be revealed at a rate of either 1, 3 or 5 seconds per card in each trial, instead of a constant rate of 3 seconds per card as in Experiment 1.

**Fig 6:**
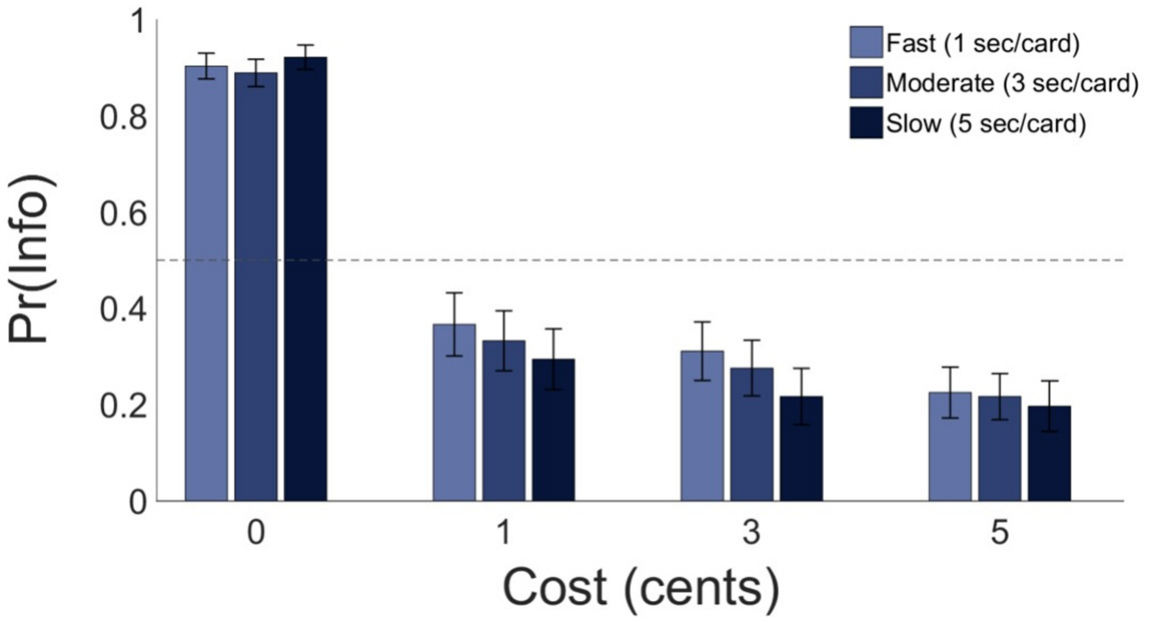
Behavioural results for Experiment 2. Mean proportion of informative stimulus choices (denoted *Pr(Info)*) as a function of information cost and information rate. Light blue bars represent the fast speed condition, medium blue bars the moderate speed condition, and dark blue bars the slow rate conditions. Error bars represent SEM. Proportions of information-seeking choices decreased as information rate slowed, particularly for positive information cost conditions (costs of 1, 3, and 5 cents). N = 40.

Participants in Experiment 2 made information-seeking choices on 42.89 percent of trials (*SD* = 24.72), indicating comparable task performance to Experiment 1. Participants showed good levels of task engagement, with low levels of missed responses (*M* = 1.11 percent, *SD* = 1.87). On average, participants sacrificed 2.75 percent of available winnings in exchange for early information about the lottery outcome (*SD* = 3.49). A 4×3 repeated-measures ANOVA was used to assess the effect of information cost (0, 1, 3, 5 cents) and information rate (1, 3, 5 seconds per card) on information-seeking choice proportions. The significant main effect of information cost on information-seeking choice proportions was replicated, (*F*(1.86, 72.70) = 84.16, *p* < .001; partial *η*^2^ = 0.68), and, crucially, we also found a significant main effect of information rate on information-seeking choice proportions (*F*(2, 78) = 3.60, *p <* .05; partial *η*^2^ = 0.08; see Fig 6). This indicates that behaviour was modulated by the rate as well as the cost of information, though the effect size of information rate was substantially smaller than the effect size of information cost. There was no significant interaction between information cost and information rate (*F*(6, 234) = 1.85, *p* = .09) although there was a non-significant trend for the effect of information rate to be larger in the positive cost conditions (1, 3, and 5 cents) than in the zero-cost condition.

One potential explanation of this effect is that participants may have preferred sooner rather than later resolution of uncertainty. To explore this possibility, we formulated and compared additional computational models assuming temporal discounting of future states. For most participants, these models did not provide a better fit to data than the undiscounted UP and EVI models. However, the degree to which participants discounted future information was associated with individual differences in the effect size of information rate (see S3 Text).

## Discussion

Participants in the present study consistently preferred an informative stimulus to a perceptually equivalent non-informative stimulus, despite the fact that information could not be used to improve future outcomes. Moreover, in many cases participants were willing to sacrifice future monetary reward in exchange for this early but non-instrumental information. Since the non-informative stimulus was always of equal or greater expected monetary reward, this pattern of results strongly suggests that participants assigned an intrinsic value to information. This stands in contrast to predictions of instrumental theories of information valuation based on expected reward maximization [10–12], but is consistent with the preference for early resolution of uncertainty posited by decision theory [16–18], and with the behavioural sensitivity to information gain proposed by active inference [19]. Although it has previously been conjectured that intrinsic valuation of information may result in willingness to pay for payoff-irrelevant information [27], this effect has not previously been demonstrated in humans using a well-controlled cognitive task.

We found that the UP model, a novel computational model of information seeking, provided a good account of intrinsic valuation of information by assuming that preference for information resulted from aversion to temporally prolonged uncertainty [13, 28–30]. Notably, we found that the model was able to capture individual differences in strength of information preference across participants, as well as providing a good account of group-level results. It is important to note that aversion to temporally prolonged uncertainty as implemented in the UP model is mathematically and conceptually distinct from the economic concept of risk aversion [32]. Risk aversion as commonly understood cannot predict the preference for information exhibited by participants in the present task, since at the point of choice informative and non-informative stimuli were associated with identical outcome probabilities, and differed only in the rate at which outcome uncertainty was resolved. Similarly, although the informative stimulus was associated with reduced payoff variance in non-zero cost conditions, a simple mean-variance tradeoff [33] does not provide a coherent account of preference for information either, since participants’ information preference was strongest in the zero-cost condition, where both mean and variance of payoffs were identical for the two stimuli. Notwithstanding this result, however, we also found that the UP model fit data well even when trials in the zero-cost condition were excluded from analysis, thus giving us confidence that participants assigned an intrinsic value to information in both zero and non-zero information cost conditions.

Furthermore, consistent with the theory that information valuation is a stable trait-level feature of individuals, we found that information preference was stable across time within the task. This would not have been expected if, for instance, participants only sought information in order to learn payoff contingencies in early blocks of the task. In addition, we found that preference for information was modulated by the rate at which uncertainty was resolved, such that participants exhibited a stronger preference for non-instrumental information when information was delivered at a faster rate. This result is analogous to the preference for faster monetary reward rate in choice behaviour [34]. Moreover, although the effect of information rate cannot be directly captured within the UP model, the direction of the information rate effect is consistent with discounting of future information, analogous to the temporal discounting of future rewards in human judgment and decision making [35]. As such, the results of the present study are in line with the proposal that humans treat information as though it has an intrinsic reward value commensurable with (and perhaps encoded in the same neural circuits as) primary and monetary reward [2, 15, 22]. However, we also note that the results of Experiment 2 demonstrated a relatively small effect size of information rate; future research should therefore further investigate the nature and robustness of this effect.

Participants in the present study sacrificed future monetary reward in exchange for early but payoff-irrelevant information. This behaviour, which is suboptimal from the perspective of expected reward maximization, has previously been observed in pigeons and starlings [6–8]. In the present study we present for the first time a well-controlled cognitive paradigm with which to assess this behaviour in humans. We note that previous studies in human participants have reported results generally consistent with a willingness to pay for early resolution of uncertainty, such as a greater preference for a risky lottery whose uncertainty was resolved immediately relative to an equivalent lottery whose uncertainty was resolved gradually [36, 37], and a willingness to pay for immediate resolution of uncertainty rather than a 50 percent probability of delayed resolution of uncertainty [38]. Among cognitive studies explicitly assessing the value of non-instrumental information, Pierson & Goodman (2014) found that participants self-reported a willingness to pay for non-instrumental information [39]. However, this behaviour was only assessed using a hypothetical survey task, which may have confounded results given the well-documented disparity in behaviour between hypothetical and incentive-compatible choice tasks [40]. Separately, a behavioural economic study using an incentive-compatible task concluded that observing non-instrumental information was related not to intrinsic valuation of information *per se*, but to a desire to increase one’s post-hoc confidence regarding an earlier decision [41]. This explanation predicts that participants will only seek non-instrumental information if it provides feedback on an earlier decision. Our results are inconsistent with this explanation, since no such decision was present in the task used in the present study. The strength of our conclusions is based on a well-controlled task in which informative and non-informative stimuli were perceptually identical, and in which preferences for information were elicited in a fully incentive-compatible fashion.

The results of the present study are also conceptually consistent with the preference for early resolution of uncertainty described in economic decision theory by the Kreps-Porteus model [16], and used to account for anomalous patterns of stock pricing in finance by Epstein and Zin [18]. Our empirical and computational findings complement these theories: whereas the Kreps-Porteus model demonstrates that preference for early resolution of uncertainty is a consequence of a particular formulation of recursive utility, in the present study we present a cognitive process model which provides a psychologically plausible account of information-seeking behaviour. Specifically, our results provide evidence that information seeking may result from an aversion to temporally prolonged uncertainty [13, 28–30]. One interesting finding in this respect was that there was a negative correlation across participants between the UP model’s information preference parameter *k* and its response stochasticity parameter *β*. This correlation was such that participants who assigned a stronger intrinsic value to information also tended to exhibit greater response stochasticity. This relationship is of theoretical interest, since it has been proposed that information-seeking behaviour may result from high levels of response stochasticity in exploration-exploitation dilemmas (e.g. [25], but see also [42]), or via *ε*-greedy action selection methods in reinforcement learning [43]. Although the superior goodness-of-fit of the UP model in the present study clearly indicates that response stochasticity alone cannot account for participants’ information-seeking choices, the correlation between *k* and *β* raises the interesting possibility that intrinsic valuation of information and response stochasticity may make separable but related contributions to exploratory behaviours. Under this hypothesis, the *k* parameter would correspond to directed exploration, a goal-directed process aimed specifically at reducing uncertainty, whereas the *β* parameter would correspond to a more diffuse form of undirected exploration. Future research should further investigate this hypothesis.

However, it is also important to note that behaviour could also be explained by an appetitive drive for information as well as an aversion to uncertainty. Because, according to information theory, uncertainty and information are mathematical conjugates [44], aversion to uncertainty makes similar behavioural predictions to an appetitive desire for information. Accordingly, it is possible to reparametrise the UP model to explain behaviour in terms of an information value bonus, rather than an uncertainty penalty, with equivalent behavioural predictions. As such, behavioural data alone may not be sufficient to distinguish between behaviour driven by uncertainty aversion and behaviour driven by an appetitive desire for information. One possibility for future research is that, since appetitive and aversive stimuli are processed in distinct neural circuits [45], it may be possible to use neural recordings to disentangle these two potential cognitive mechanisms for information valuation.

In accounting for the results of the present study we have primarily drawn upon theories proposing that intrinsic valuation of information can result from an aversion to temporally prolonged uncertainty [18, 28–30]. However, alternative theoretical frameworks can also account for the present study’s results in terms of a positive information bonus (consistent with an alternate parametrisation of the UP model described below). For instance, it has been proposed that agents may derive utility from maintaining an internal model of the environment which is well-adapted to the statistics of natural stimuli [46, 47]. A natural consequence of this model is that agents should place a non-zero value on information about the external environment, even when no behaviour can be directly conditioned on this information (for instance, an intrinsic curiosity reward, as proposed by Schmidhuber, 2009 [48]). Similar intuitions regarding the appetitive value of information have been formalised in several general theories of cognition, including active inference theory [19, 47] and optimal Bayesian exploration [49]. Such theories can be extended to account for seemingly paradoxical attitudes towards information in other settings, such as participants’ preference for maximising entropy over choice options as well as simply maximising expected reward [50], as well as seemingly paradoxical patterns of self-deception in financial choices [51]. At a neurocomputational level, appetitive valuation of information is also consistent with the notion of dopaminergic novelty or exploration bonuses [52].

Notwithstanding the above, however, a further possibility proposed by Beierholm and Dayan (2010) is that an apparent preference for informative stimuli might, in fact, be driven by task disengagement, leading to a relatively greater decrease in the subjective value of the non-informative stimulus [53]. The paradigm tested in the present study sought to prevent such task disengagement by means of pseudo-randomly occurring ‘catch trials’, in which participants were required to make a rapid button-press response to one of the cards in either the informative or the non-informative stimulus. This manipulation helped to ensure that participants maintained task engagement even when observing the non-informative stimulus. Although this cannot conclusively rule out the possibility that participants were somewhat more engaged by the informative than the non-informative stimulus, it does ensure that participants could not fully disengage from the task during observation of non-informative stimuli. Moreover, the behavioural paradigm that we tested allows for well-controlled manipulation of task engagement: by increasing or decreasing the frequency of catch trials, it should be possible to manipulate the degree to which participants disengage during viewing of the non-informative stimulus. The Beierholm and Dayan model makes the empirical prediction, which could be tested in future research, that information preference ought to be strongest for greater degrees of task disengagement (that is, low catch trial frequency), and that information preference ought to decrease in strength with increasing catch trial frequency.

These theoretical caveats notwithstanding, the results of the present study provide clear behavioural evidence that human participants derive utility from non-instrumental information in a manner inconsistent with traditional models of information valuation. The behavioural task assessed in the present study provides a well-controlled means for assessing intrinsic valuation of non-instrumental information, and the UP model allows for individual differences in the strength of information valuation to be quantified in a principled and mathematically tractable fashion.

Since intolerance of uncertainty has been proposed as a trans-diagnostic treatment marker for emotional disorders [54], understanding information-seeking behaviours may shed light on the symptomatology of disorders including generalised anxiety disorder and obsessive compulsive disorder [55]. For instance, the compulsive checking behaviours which are a hallmark of obsessive compulsive disorder may represent a form of pathological information-seeking behaviour. From this perspective, it might be possible to redescribe some behavioural features of obsessive compulsive disorder as an excessive intrinsic valuation of information driven by excessive levels of aversion to uncertainty. As such, we would hypothesise that individuals with obsessive compulsive disorder would exhibit a high willingness to pay for non-instrumental information in the task used in the present study.

The results of the present study also have bearing on studies of the exploration-exploitation dilemma, in which participants trade off information seeking and reward seeking [25]. A common finding in this literature is that participants seek out more information than is optimal [26]. Our results may help shed light on this finding: intrinsic valuation of information may cause participants to place a premium on information, resulting in a valuation of information in excess of its purely instrumental value. More broadly, we note that although preference for information in the present task was suboptimal from the restricted perspective of monetary reward maximisation, intrinsic valuation of information may be adaptive in more naturalistic environments. Choices in natural settings often resemble dynamic constrained optimisation problems, in that organisms are presented with epistemic uncertainty and poorly defined action-outcome contingencies. In these environments, the instrumental value of seeking information may be computationally intractable, and intrinsic valuation of information might induce a bias toward gathering information that encourages exploratory behaviour even when the usefulness of that exploratory behaviour is not immediately clear. As such, intrinsic valuation of information may induce patterns of behaviour akin to an exploration or novelty bonus [52]. Therefore, in dynamic and uncertain environments intrinsic valuation of information may be associated with profound long-run benefit, in spite of locally suboptimal outcomes in artificial task environments such as that employed by the present study. More broadly, we do not propose that there exists any single level of intrinsic information valuation that will produce optimal behaviour across all environmental conditions. For instance, a strong intrinsic valuation of information may be beneficial when exploration costs are low and overall uncertainty is high, but result in suboptimal performance in situations where exploration is relatively expensive, or where overall environmental uncertainty is low.

In summary, our results provide strong evidence for intrinsic valuation of information in humans, and we present a novel cognitive process model which suggests that aversion to prolonged uncertainty may be an important psychological determinant of this value. We show that intrinsic valuation of information can result in seemingly suboptimal behaviours, such as a willingness to sacrifice future monetary reward in exchange for immediate but unusable information about relevant future outcomes. More broadly, our results provide a plausible psychological mechanism for human curiosity and exploration, and may explain features of decision making under uncertainty that have hitherto been considered irrational.

## Materials and Methods

### Participants

Participants were staff and students of the University of Melbourne. In Experiment 1, we recruited forty-one participants (15 male, 26 female; 40 right-handed, 1 left-handed), aged 18 to 31 (*M* = 22.28, *SD* = 2.63). In Experiment 2, we recruited 40 participants (14 male, 26 female; all right-handed) aged 18 to 32 (*M* = 22.90, *SD* = 4.04). Participants gave voluntary informed consent, research was conducted in accordance with the Declaration of Helsinki, and protocols were approved by the University of Melbourne Human Research Ethics Committee (ID 1341084). As compensation for participation, participants received a flat payment of AUD $10 plus all lottery winnings (lottery winnings in Experiment 1: *M* = $9.10, *SD* = $1.86; Experiment 2: *M* = $7.15, *SD* = $0.91).

### Procedure

Stimuli were presented using the Psychophysics Toolbox [56] and MATLAB R2012b (The Mathworks, Natick, MA) on a Macintosh Mini connected to an LCD monitor with resolution 1920×1080 pixels at a screen refresh rate of 60Hz.

Experiment 1 comprised seven blocks, each consisting of sixteen trials total: four trials in each of the four cost conditions (0, 1, 3, 5 cents), with win probabilities pseudo-randomised to ensure that win rates for each cost condition were identical. As is standard practice in computational modelling studies, participants were randomly assigned to one of four pre-generated trial sequences. Participants completed the task in approximately 1 hour. In Experiment 2, the rate at which cards were revealed differed between blocks. In each block, information could be revealed at a rate of either 1, 3, or 5 seconds per card. As such, the lottery delay period varied across blocks (6 seconds total in 1 sec/card blocks, 18 seconds for 3 sec/card blocks, 30 seconds for 5 sec/card blocks). Participants completed 6 blocks of 12 trials each. Each participant was assigned to one of three counterbalanced trial orders, in which no two adjacent blocks belonged to the same information rate condition. Participants completed the task in approximately forty minutes. The primary dependent variable *Pr(Info)* was the proportion of all choices (excluding missed responses) in which participants elected to observe the informative stimulus.

To ensure that participants maintained task engagement and attended to each stimulus type equally, approximately 10 per cent of all trials were designated as *catch trials*. In catch trials, instead of revealing a black or red card, one of the cards was revealed to be a white X, to which participants responded by pressing any key within 1.5 seconds. A successful response led to progression to the subsequent trial without penalty; failure to respond resulted in a $1 penalty. Participants who failed to respond to more than two catch trials across the experiment were excluded from all further analyses. This resulted in the exclusion of one participant in Experiment 1 (successful catch trial responses in Experiment 1: *M* = 96.88%, *SD* = 5.56%; Experiment 2: *M* = 97.5%, *SD* = 5.80%). Rates of successful responses to catch trials did not differ significantly between informative and non-informative stimuli (Experiment 1: *t*(37) = 0.74, *p* = .46; Experiment 2: *t*(37) = 0.61. *p* = .55). There is therefore no evidence to suggest that participants’ catch trial performance differed as a function of stimulus type.

### Computational models

**Fig 7:**
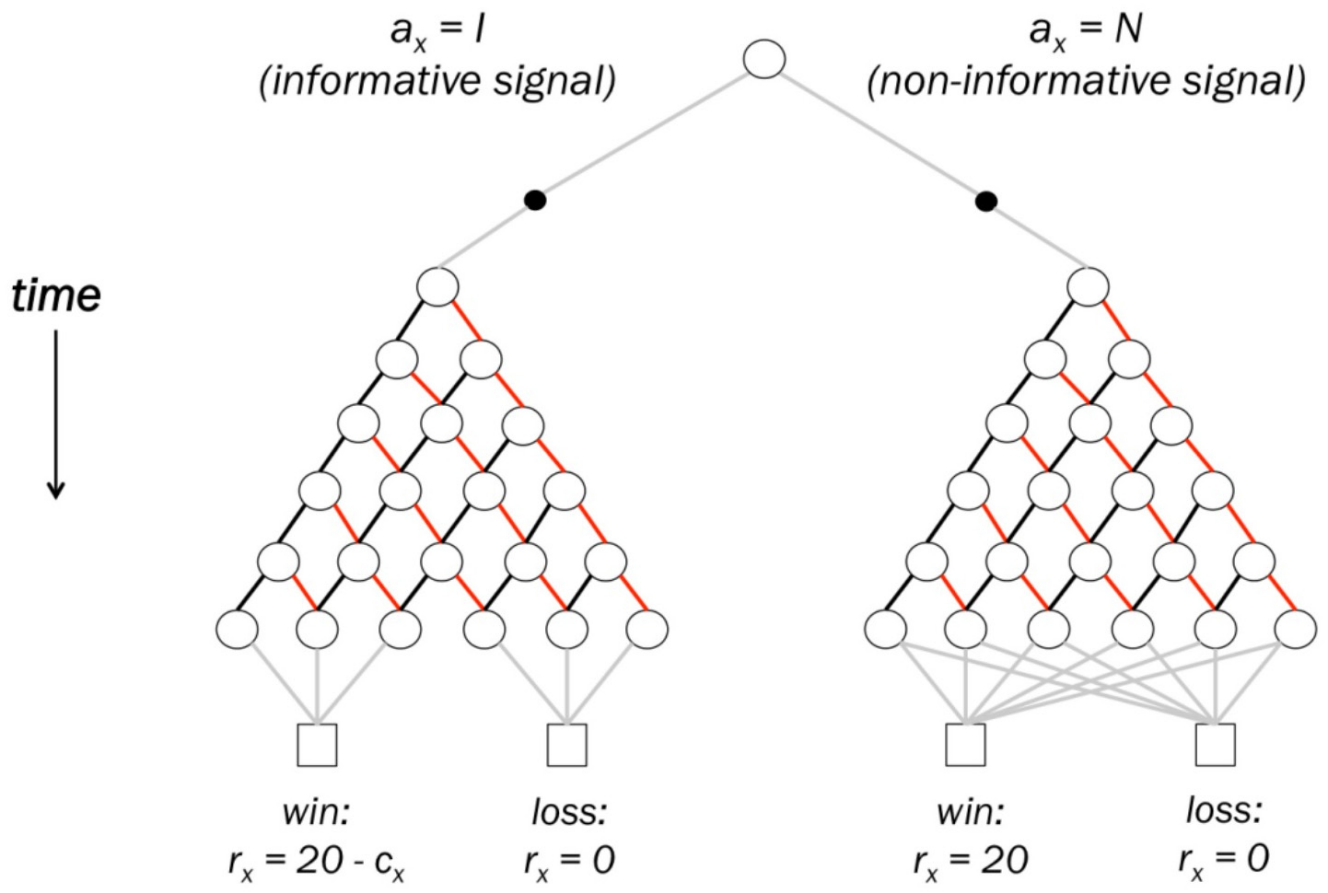
Trial structure represented as a Markov Decision Process. Open circles represent states, filled circles represent actions, and squares represent terminal (trial-end) states. Lines connecting states represent state transitions; implicit in the above representation is that where no lines connect two states, the transition probability between these states is zero. Participants begin each trial in the topmost state, and can make one of two actions: to observe the informative signal (left filled circle), or to observe the non-informative signal (right filled circle). Following this choice, participants move stochastically through one of two state trees, representing the two signal types. Within each tree, transition probabilities are defined by the relative probabilities of drawing black and red cards (transitions represented respectively by black and red lines). State transitions which do not involve drawing a card are indicated in grey. The structure of the two signal trees is identical, with the exception that in the informative signal tree, each of the six states which can result after all cards are drawn (bottom row of circles) transitions to one of the two possible lottery outcomes (win/loss) with probability 1. In the non-informative signal tree, states after all cards are drawn may transition to either of the two possible outcomes.

Models represented the task as an MDP, in which each trial was a decision problem with two actions (informative/non-informative stimuli), discrete states corresponding to different configurations of red and black cards (see Fig 7), and state transition probabilities corresponding to relative probabilities of red/black cards. Using dynamic programming, we calculated the action value of observing each of the two stimuli under varying assumptions about the nature of valuation, and used these action values to predict choice proportions. Competing models used identical MDP task representations, and differed only in the definition of the equation used to calculate action values.

The information seeking task used in the present study can be formally characterised as follows: in each trial *x*, participants chose an action *a*_*x*_ from the set *A* = {*I*, *N*}, where *I* denotes a choice to observe the informative signal and *N* denotes a choice to observe the non-informative signal. The outcome of each trial was denoted *y*_*x*_ and could be either a win, *y*_*x*_ = 1, with probability *P*_*black*_, or a loss, *y*_*x*_ = 0, with probability *P*_*red*_. By definition, *P*_*red*_ = 1—*P*_*black*_, and in the present study, it was always the case that *P*_*black*_ *= P*_*red*_ *=* 0.5. Let *c*_*x*_ denote the cost in cents of observing the informative signal on a given trial, drawn from the set *C* = {0, 1, 3, 5}. Each trial’s winnings, denoted *r*_*x*_, depended only on the action selected and the predetermined outcomes of the trial lottery, such that

$$r_{x}=\{\begin{array}{ll} 20-c_{x}, & a_{x}=I\;and\;y_{x}=1\\ 20, & a_{x}=N\;and\;y_{x}=1\\ 0, & otherwise \end{array}$$ (1)

The task’s structure was implemented as an MDP by considering every different possible configuration of red and black cards as a separate state. This is a natural way of discretising trials of the task such that each state represents a perceptually distinct epoch within the trial. The general structure can be described as follows: depending on the action selected, a participant traverses one of two state trees, corresponding to the two signal types. As such, the two state trees are structurally identical and differ only in the sense that the final card state reached in the informative signal tree perfectly predicts whether the lottery outcome will be a win or a loss, whereas the final card state in the non-informative signal tree may transition to either outcome. Since transitions within the state trees depend on the relative likelihood of drawing red and black cards, state transitions are governed by the probabilities *P*_*black*_ and *P*_*red*_. This structure is illustrated schematically in Fig 7.

The structure and parameters described above give a complete description of the states, actions, rewards, and state transition probabilities of the information seeking task. As a result, standard analytic techniques of MDPs can be applied to solve this decision problem. Specifically, using dynamic programming [57], it is possible to calculate the action value *Q* of each of the actions *I* and *N*, and to use these action values to predict choice proportions for the two actions. Each of the competing behavioural models used an identical MDP framework, and models differed only in how the action values are calculated. More precisely, in each of the models assessed, action values were calculated by solving a Bellman optimality equation, where only the definition of this equation and its free parameters varied between models. Model fitting procedures were identical for each of the models.

#### Expected Value of Information (EVI) model

The EVI model assumed that agents consider solely the instrumental value of information [11]. As such, the state value equation for this model is simply the standard recursive Bellman optimality equation for stochastic programming in MDPs [57]:

$$V\left(s\right)={\underset{}{\text{max}}}_{a}{\sum_{}}_{s\prime }Pr(s^{\prime }|s,a)\left[R\left(s^{\prime },s,a\right)+V\left(s^{\prime }\right)\right]$$ (2)

This equation can be interpreted as a probability-weighted sum over future rewards and states, where *a* represents an action taken in state *s*, causing a transition to a successor state *s′* with probability *Pr*(*s*′|*s*, *a*), with concurrent receipt of a transition reward *R*(*s*′, *s*, *a*) (corresponding to monetary payoffs in the present task). The value of the successor state *s′* is denoted *V*(*s*′) and is itself also calculated recursively according to Eq 2. As such, the action-value equation for this model was simply the standard recursive Bellman optimality equation for stochastic programming in MDPs:

$$Q\left(s,a\right)=\Sigma_{s\prime }Pr(s^{\prime }|s,a)\left[R\left(s^{\prime },s,a\right)+V\left(s^{\prime }\right)\right]$$ (3)

The quantity *Q*(*a*) represents the value of taking the action *a* in state *s*, causing a transition to a successor state *s′* with probability *Pr*(*s*′|*s*, *a*), with concurrent receipt of a transition reward *R*(*s*′, *s*, *a*) (corresponding to monetary payoffs). Note that since there was only one state in the present study from which actions could be taken (see Fig 7), we henceforth use the shorthand *Q*(*a*) to denote the value of the action *a*.

An implicit assumption of the EVI model is that information is only valuable to the extent that it can be used by agents to increase future expected reward; that is, agents should only seek information when it increases their expected reward. This follows from the fact that state values in Eq 2 are calculated solely on the basis of the monetary reward matrix *R*. In a task such as that used in the present study, where information and expected reward are orthogonal, this model predicts that participants should display no preference for information. Specifically, the EVI model’s prediction is that when there is no cost placed on observing the informative signal participants should be indifferent between the two signals, and that when any non-zero cost is placed on the informative signal, participants should prefer observing the non-informative signal.

In describing the task as an MDP, we have adopted a somewhat different framework for modelling the structure of the decision to standard decision analysis. However, for the model presented above, the value of observing the informative signal *Q*(*I*) closely resembles the decision-analytic quantity termed the Expected Value of Sample Information [EVSI; 11]. In decision analysis, the EVSI is theoretically always non-negative, since information can either increase future expected reward (in which case EVSI > 0) or not alter future expected reward (in which case EVSI = 0). In the task used in the present study, observing the informative signal had an EVSI equal to 0, since observing information could not increase future expected reward. When the cost of observing the informative signal was also considered, this meant that observing the informative signal always had a EVSI equal to zero (for *c* = 0), or less than zero (for *c* > 0).

#### Uncertainty Penalty (UP) model

The UP model was a hierarchical extension of the EVI model, and assumed that as well as seeking to maximise expected reward, agents were averse to the presence of uncertainty over time [29]. This aversion was implemented in UP model as a penalty of all states *s* of the MDP according to the relative probabilities of winning and losing from state *s*: *P*_*win*_(*s*) and *P*_*loss*_(*s*). We defined this penalty function *H*(*s*) to be the binary entropy function from information theory, since this function is zero in the case of complete certainty and maximal in the case of equal probabilities of winning and losing:

$$H\left(s\right)=\left(-P_{win}\left(s\right){\text{log}}_{2}P_{win}\left(s\right)\right)-\left(P_{loss}\left(s\right){\text{log}}_{2}P_{loss}\left(s\right)\right)$$ (4)

The state value equation for the UP model is created by incorporating this penalty function into normative value equation of the EVI model via an additional exponential term:

$$V\left(s\right)={\text{max}}_{a}\Sigma_{s\prime }Pr(s^{\prime }|s,a)\left[R\left(s^{\prime },s,a\right)+V\left(s^{\prime }\right)e^{-kH\left(s^{\prime }\right)}\right]$$ (5)

Eq 5 describes the value of different states in the UP model; as such, this equation is functionally equivalent to Eq 2 from the EVI model. However, these two equations differ in that Eq 5 penalises the values of successor states according to their outcome uncertainty (which, in turn, is calculated using Eq 4). Importantly, Eq 5 allowed for the strength of the uncertainty penalty to vary between participants according to a participant-specific scaling parameter *k*. When *k* is equal to 0, the exponential term in Eq 5 is equal to one, meaning that states’ values are unaffected by outcome uncertainty; in this case, the UP model reduces to the EVI model. For *k* > 0, an uncertainty penalty is applied to all states. Since the informative stimulus reduces uncertainty faster and in more states than the non-informative stimulus, non-informative states are penalised more heavily than informative states, inducing a preference for observing the informative stimulus. For *k* < 0, the converse is true: an uncertainty bonus applies to all states, inducing a preference for the non-informative stimulus. The UP model therefore predicts information seeking from the fact that, although monetary reward is received at the same time for each stimulus, the informative stimulus is associated with less time spent in uncertain states. Given this framework, action values for the UP model can then be calculated as:

$$Q\left(a\right)=\Sigma_{s\prime }Pr(s^{\prime }|s,a)\left[R\left(s^{\prime },s,a\right)+V\left(s^{\prime }\right)e^{-kH\left(s^{\prime }\right)}\right]$$ (6)

As an aside, we note that although the UP model used in the present study quantifies information valuation in terms of aversion to states’ uncertainty, there exist alternative specifications of the action-value equation which can produce equivalent quantitative predictions regarding behaviour. In particular, it is possible to reparametrise Eq 6 such that intrinsic valuation of information is expressed in terms of value bonus for information, rather than an aversion to states’ uncertainty (see also Sun, Gomez & Schmidhuber, 2011 [49] for a discussion of conceptual differences between information reward signals and standard reinforcement learning rewards). This is because moving from one uncertain (and therefore aversive) state to a less uncertain (and therefore less aversive) state is mathematically equivalent to receiving an information-related value bonus during the state transition. This value bonus will be equal to the amount of uncertainty which has been reduced in the transition, scaled by an individual information preference parameter:

$$Q\left(a\right)=\Sigma_{s\prime }Pr(s^{\prime }|s,a)\left[R\left(s^{\prime },s,a\right)+\hat{k}.I\left(s^{\prime },s,a\right)+V\left(s^{\prime }\right)\right]$$ (7)

where $\hat{k}$ is a reparametrised information scaling parameter, and *I*(*s*′, *s*, *a*) is an information value bonus quantified as the difference in entropy of beliefs following a state transition, as follows:

$$I\left(s^{\prime },s,a\right)=H\left(s^{\prime }\right)-H\left(s\right).$$ (8)

Notwithstanding the above, all model fits and reported parameter values in the present study are calculated using the uncertainty-aversion parametrisation of the UP model (Eq 6), not the information-bonus parametrisation (Eq 7).

#### Model fitting procedure

Given that the two models described above share a common MDP structure, it is possible to specify an overall choice rule and likelihood estimation procedure that is independent of how each individual model calculates action values. For every cost condition *c*, each of the models supplies a state-action value for observing the informative signal, *Q*_*c*_(*I*), and a state-action value for observing the non-informative signal, *Q*_*c*_(*N*). A mapping from these model-derived action values to informative signal choice probability can be accomplished separately for each cost condition using a “softmax” or Luce choice rule:

$$P_{c}\left(I\right)=\frac{e^{\beta Q_{c}\left(I\right)}}{e^{\beta Q_{c}\left(I\right)}+e^{\beta Q_{c}\left(N\right)}}$$ (9)

In this equation, *β* is an inverse temperature parameter, *β* ≥ 0, governing the determinism of choices, and is constant across different cost conditions. As the value of *β* increases, agents deterministically choose the action with the higher action value; for *β* = 0, choices are unrelated to action values, and all actions are equally likely to be selected. Applying Eq 9 with the action value of the non-informative signal in the numerator, it is also possible to show that *P*_*c*_(*N*) = 1 –*P*_c_(*I*).

**Fig 8:**
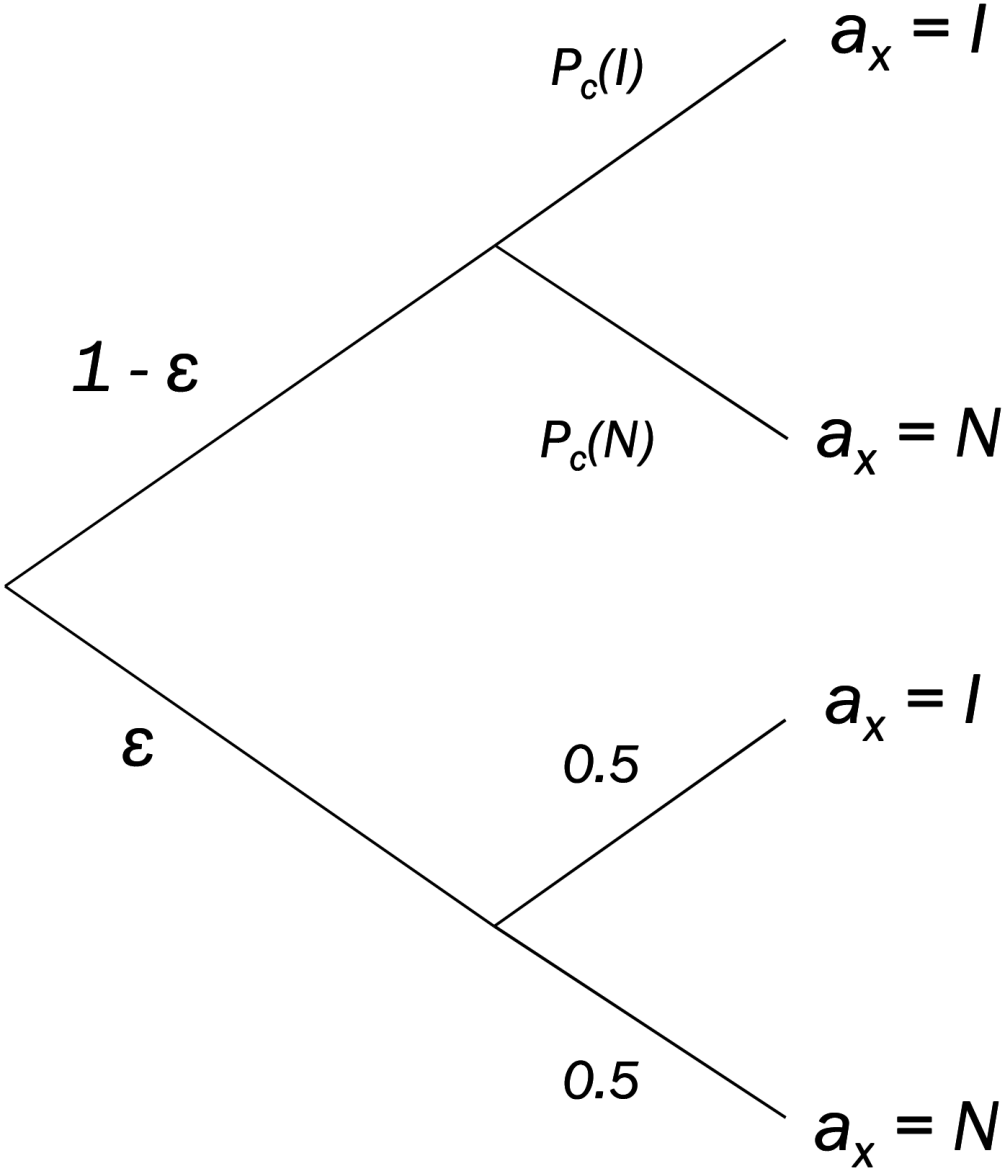
Latent mixture model of choice probability. In the present study, choice probabilities were assumed to be a latent mixture of erroneous button presses (with probability *ε*) and accurate button presses (with probability 1−*ε*). Since erroneous button presses are by definition undirected, these choices are therefore equally likely to result in the selection of the informative signal or the non-informative signal. This has the effect of placing a floor of ε2 and a ceiling of 1−ε2 on choice probabilities for each option.

All models used in the present study also included an additional parameter representing the probability of making an incorrect button press. The rationale for including this additional parameter was that during post-task debriefing, some participants who otherwise behaved in a relatively deterministic fashion reported making one or more mistaken button press because of the task’s randomised response mapping. While one practice is to account for errors of this kind by the softmax equation’s *β* parameter, when responses are otherwise strongly deterministic, a single mistaken button press can substantially affect model likelihood (for instance, when a participant mistakenly selects the informative signal in a condition where the model’s predicted probability of this action would otherwise have been at or near zero). To overcome this issue, choice probabilities in the models presented above were represented by a latent mixture model (Fig 8). In this latent mixture model it was assumed that on any given trial there was a small but non-zero probability *ε* that a participant would make a mistaken button press. The parameter *ε* was fit as a free parameter across participants. However, in order to ensure that our results were not compromised by this assumption, we also performed a control analysis assessing model fits for *ε* = 0, corresponding to the assumption that participants made no erroneous button presses (see S2 Text).

Therefore, *Pr*(*a* = *I* | *c*), the overall choice probability for observing the informative set in the cost condition *c*, is given by simple probability calculus (note that *Pr*(*a* = *I* | *c*) = *P*_*c*_(*I*) for *ε* = 0):

$$Pr\left(a=I|c\right)=0.5\varepsilon +P_{c}\left(I\right)\left(1-\varepsilon \right)$$ (10)

Since there were only two actions available to participants on each trial, the probability *Pr*(*a* = *I*) can be interpreted as a binomial rate parameter. Each model’s overall likelihood can therefore be calculated as a product of binomial likelihoods across the four cost conditions in the set *C*:

$$L=\prod_{i=1}^{4}\left(\begin{array}{c} n_{i}\\ m_{i} \end{array}\right)\;Pr{\left(a=I|C_{i}\right)}^{m_{i}}{\left(1-Pr\left(a=I|C_{i}\right)\right)}^{n_{i}-m_{i}}$$ (11)

Where *n*_*i*_ represents the number of trials in cost condition *C*_*i*_ and *m*_*i*_ represents the number of times the informative signal was observed in cost condition *C*_*i*._ Trials in which participants did not record a response were excluded.

All models were fit with maximum-likelihood estimation, using the interior point algorithm as implemented in MATLAB R2015b (The Mathworks, Natick, MA).

## Supporting Information

S1 Text UP model parameters. (PDF)

S2 Text Fit and interpretation of *ε* parameter. (PDF)

S3 Text Model fits for Experiment 2. (PDF)

S1 Fig *k* parameter and behavioural data. (TIFF)

S2 Fig *β* parameter and behavioural data. (TIFF)
