# Supplementary material for: Intrinsic Valuation of Information in Decision Making under Uncertainty
Source: PLoS Comput Biol. 2016 Jul 14;12(7):e1005020. doi: 10.1371/journal.pcbi.1005020 (PMC4944922; doi:10.1371/journal.pcbi.1005020)
Supplement: S1 Text — (PDF) [file pcbi.1005020.s001.pdf]

1. UP model parameters

Given that the UP model provided the best fit to data of the models assessed, we next sought to determine the psychological interpretation of the k parameter indexing the strength of the uncertainty penalty applied to states of the MDP. Theoretically, we can suggest that participants with a higher fit value of k are those for whom the presence of uncertainty over time is more aversive. This aversion tends to affect the non-informative signal more strongly than the informative signal, since uncertainty is reduced in more card states of the informative signal than of the non-informative signal. We would therefore expect that participants with a stronger uncertainty penalty would make more information-seeking choices.

To test that this was the case, we used a Spearman correlation coefficient to assess the strength of the relationship between the k parameter and proportion of information-seeking choices across participants. We found a strong positive correlation (ρ = 0.95, p < .001), indicating that participants made more information-seeking choices as the strength of the uncertainty penalty increased (see Figure S1a). This confirms that empirical fit values of the k parameter behaved as predicted by theory. In terms of choice behaviour, the effect of this penalty is to induce a bias toward observing the informative signal, where this bias increases in strength for increasing values of k. In addition, we also observed a significant negative correlation between the β parameter and proportions of information-seeking choices (ρ = -.33, p = .04), indicating that participants with greater response stochasticity (lower β values) tended to make more information-seeking choices (see Figure S2a).

Figure S1a reveals that the relationship between fit values of k and overall proportion of information-seeking choices was positive and generally log-linear. However, close inspection reveals four cases which were outliers from this general pattern. These were participants with a relatively large fit value of k (greater than 1)—indicating a strong uncertainty penalty—who

made relatively fewer information-seeking choices than expected. Response patterns for these participants across cost conditions are presented in Figure S1b. It can be observed that whereas for the majority of participants information choice proportion was decreasing in the cost of information, these four participants displayed either an increasing rate of information choice as information cost increased, or a non-monotonic relationship between information choice proportion and information cost. This suggests that these participants may be outliers in the plot in Figure S1a because they adopted a degenerate choice strategy. In support of this proposition, we observe that the UP model as presented here is incapable of predicting choice proportions which increase or are non-monotonic as a function of information cost.

Overall, the median fit value of k across participants was 0.03 (interquartile range = 0.09), and the median fit value of β was 11.81 (interquartile range = 71.04). Both parameters displayed a very large positive skew as a result of the four outlier participants described above. Results showed a non-significant trend toward a negative relationship between k and β (ρ = -.27, p = .09; see Figure S2b). This relationship was significant when the four outlier participants were excluded (ρ = -.56, p = .00004), indicating that participants with a stronger intrinsic valuation of information (higher k values) tended to have relatively higher response stochasticity (lower β values).
