# Supplementary material for: Intrinsic Valuation of Information in Decision Making under Uncertainty
Source: PLoS Comput Biol. 2016 Jul 14;12(7):e1005020. doi: 10.1371/journal.pcbi.1005020 (PMC4944922; doi:10.1371/journal.pcbi.1005020)
Supplement: S2 Text — (PDF) [file pcbi.1005020.s002.pdf]

2. Fit and interpretation of ε parameter

The error rate parameter ε was fit using maximum likelihood estimation, and was fixed to take the same value across participants. It was found that the best-fitting model had an ε value of 0.0526, corresponding to approximately 5 per cent probability of an erroneous button press on each trial.

We next wished to ascertain that the relative performance of the EVI and UP models was not dependent on the parameterisation of the task as a mixture model of with erroneous button press rate ε. To this end, we re-ran all model analyses under the more restrictive assumption that participants did not make erroneous button presses (i.e. with the parameter ε set to zero). Table S1 shows that the relative performance of the models was not materially altered when this restriction was applied, indicating that the results reported in the main text above for $\varepsilon = 0.0526$ still hold for a non-latent-mixture model.

Table S1. Behavioural model fits for 4405 choices by 40 participants ($\varepsilon = 0$)

Model	Free parameters (per participant)	*-LL*	*BIC*	McFadden's R^2	Δ*BIC*	*n* best fit
EVI	1	975.98	2287.58	0.49	75.45	6
UP	2	411.76	1494.76	0.78	285.36	34

-LL: negative log-likelihood. *BIC*: Bayesian Information Criterion. Δ*BIC* represents the change in BIC from models fit with $\varepsilon = 0.0526$ to models fit with $\varepsilon = 0$; positive values indicate a poorer model fit for $\varepsilon = 0$. Numbers in the *n* best fit column are based on a comparison of individual-participant BIC values for each model.

In contrast to many commonly used decision tasks, where participants' choices are quite noisy, many participants in the present study made very deterministic choices (e.g. always choosing information if free; always choosing no information for positive information cost). It is in this particular context that the ε parameter proves valuable: when choices are

highly deterministic, even a single mistaken button press can result in very different parameter estimates. There were several such cases among participants completing Experiment 1, and it is primarily among these participants that including an ε parameter improved model fits. As such, we believe that including an ε parameter meant that model estimates of the k parameter more accurately estimated the strength of individual participants' information preference.

On a single-participant level, both the β and the ε parameter are able to account for response stochasticity. This fact motivated the modelling decision to constrain the ε parameter across participants, rather than allowing it to vary freely like the β. If we had allowed both ε and β to vary freely across participants, the models would have become unidentifiable, since individual-participant variance related to response stochasticity could have been equally accounted for by either ε or β. On the other hand, by fitting ε at the group-level, we ensured that our parameter estimates were fully identifiable.
