# Supplementary material for: Intrinsic Valuation of Information in Decision Making under Uncertainty
Source: PLoS Comput Biol. 2016 Jul 14;12(7):e1005020. doi: 10.1371/journal.pcbi.1005020 (PMC4944922; doi:10.1371/journal.pcbi.1005020)
Supplement: S3 Text — (PDF) [file pcbi.1005020.s003.pdf]

3. Model fits for Experiment 2

Supplementary method for discounted models

Overview of models. As well as the EVI and UP models assessed in Experiment 1, in Experiment 2 we tested two additional models. These additional models were extensions of the EVI and UP models to include an additional temporal discounting parameter; the additional models were therefore respectively classed as Discounted Expected Value of Information (DEVI) models and Discounted Uncertainty Penalty (DUP) models.

The DEVI and DUP models allowed us to assess whether the timing of future outcomes (including resolution of uncertainty, in the case of DUP models) affected decisions to seek information. Whereas the basic EVI and UP models fit to data from Experiment 1 predict that behaviour ought to be constant across the three information speed conditions assessed in Experiment 2, the DUP models predict that participants ought to show a greater preference for observing the informative set for fast information rates than for slow information rates. Intuitively, this prediction arises from the fact that uncertainty is resolved faster if cards are turned over more rapidly; participants who discount future information should therefore display a greater preference for information when it is revealed more quickly.

Discounted Expected Value of Information (DEVI) model. The DEVI model was the same as the EVI model in Experiment 1, but included an additional temporal discounting parameter γ, $0 \leq \gamma \leq 1$. The action value equation for the DEVI model is given by:

$$Q(a) = \sum_{s'} Pr(s'|s,a)\,[R(s',s,a) + \gamma^t V(s')] \qquad \text{(S1)}$$

Where t is equal to the duration (in seconds) of each state (t = 1 for the fast information rate condition, t = 3 for the moderate rate condition, and t = 5 for the slow rate condition). For $\gamma <$ 1, this model assumes that participants prefer to move more quickly into valuable states. Since

this model is an extension of the information-indifferent EVI model, discounting induces a preference with respect to time, but does not induce a preference with respect to information. When $\gamma = 1$, this model reduces to the EVI model.

Discounted Uncertainty Penalty (DUP) model. Analogous to the EVI and DEVI models, the DUP model was the same as the UP model with the exception of the additional temporal discounting parameter γ. Testing the DUP model allowed us to assess whether participants behaved as though they discounted future information as well as future reward. The action value equation for the DUP model is given by

$$Q(a) = \sum_{s'} Pr(s'|s,a)\left[R(s',s,a) + \gamma^t V(s')e^{-kH(s')}\right] \tag{S2}$$

When $\gamma = 1$, this model reduces to the UP model; when $\gamma = 1$ and $k = 0$, this model reduces to the ER model.

Model Fitting Procedure. The MDP structure of Experiment 2 was the same as that of Experiment 1, with the sole exception that states also had a variable duration t drawn from the set $T = \{1, 3, 5\}$. The model likelihood function for Experiment 2 is therefore given by a product of probabilities across i information cost conditions and j information rate conditions, analogous to the model likelihood for Experiment 1 given by Equation 11.

$$L = \prod_{j=1}^{3}\prod_{i=1}^{4}\binom{n_{i,j}}{m_{i,j}} Pr\big(a = I \mid C_i, t_j\big)^{m_{i,j}}\left(1 - Pr\big(a = I \mid C_i, t_j\big)\right)^{n_{i,j}-m_{i,j}} \tag{S3}$$

All other procedural details of model fitting were as in Experiment 1.

Supplementary results for discounted models

Model Fits. As in Experiment 1, in order to determine which computational model provided the best account of participants' choices, we calculated overall and participant-specific BIC. Relevant statistics for this comparison are presented in Table S2.

Table S2. Behavioural model fits for 2848 choices by 40 participants

Model	Free parameters (per participant)	*-LL*	*BIC*	McFadden's R^2	*n* best fit
EVI	1	889.75	1950.11	0.45	5
UP	2	390.33	1121.89	0.75	33
DEVI	2	889.16	2119.55	0.45	0
DUP	3	380.11	1272.05	0.77	2

-LL: negative log-likelihood. *BIC*: Bayesian Information Criterion. Numbers in the *n* best fit column are based on a comparison of participant-specific BIC values for each model.

Results indicated that the undiscounted uncertainty penalty model (UP) provided the best overall account of participants' choices, as well as being the best-fitting model for a substantial majority of individual participants. Likewise, the DUP model clearly outperformed the DEVI model, indicating that the inclusion of an uncertainty penalty improved models' performance even when temporal discounting was taken into account.

Relationship between Behavioural Data and Model Parameters. Interestingly, given the significant main effect of information rate, inclusion of a discount parameter did not lead to an improvement in model performance as measured by BIC (when measured by McFadden's R^2, inclusion of a discounting parameter led to a marginal improvement in model fit). In other words, the overall best-fitting model as measured by standard model comparison statistics was

not one which could account for the effect of information rate. This strongly suggests that the effect of information rate was sufficiently small in Experiment 2 that inclusion of an additional temporal discounting parameter in the DUP models did not account for a large proportion of behavioural variance in the model comparison, even though it did allow the model to account for the small decrease in information choice proportions for slower information rates.

We next examined individual participant model fits to determine whether the above interpretation was supported. If temporal discounting of information was the mechanism which produced the main effect of information rates, as hypothesised, then we would expect fit values of the DUP models' discounting parameter γ to be related to the strength of the information rate effect in individual participants. In order to assess the strength of this effect, we used linear regression to predict informative choice proportions as a function of information rate (1, 3, 5 sec/card) in individual participants. The resulting beta parameter was an index of the strength of the rate effect in individual participants as a function of information rate, with a zero beta indicating that participants showed no effect of information rate on choice behaviour, and more negative beta values indicating greater effect of information rate.

Correlation analysis revealed that beta slope values showed a significant positive relationship with the best-fitting values of the γ parameter in the DUP-log model ($r(40) = .48$, $p < .01$), indicating that participants who showed a stronger effect of information rate on choices were best fit by a model with a stronger temporal discounting parameter. In addition, we can measure the degree to which the UP model outperformed the DUP-log model for individual participants by taking a difference of participant-specific BIC values. Using a second correlation analysis, we found that beta slope values were related to the difference in model BIC values ($r(40) = -.48$, $p < .01$). This result demonstrates that the relative goodness-of-fit of the discounted model was strongly related to the degree to the strength of the information rate main effect in individual participants. These two complementary correlation analyses support

the contention that fit values of the temporal discounting parameter were related to the degree to which participants showed an effect of the information rate manipulation. We can further speculate from these results that the DUP models might outperform the undiscounted UP model in an experimental paradigm with a larger disparity in information rate conditions (e.g. 10, 30, and 50 seconds/card rather than 1, 3, and 5). Slower information rates such are likely to be more comparable to the timescales of uncertainty resolution in naturalistic settings than the three conditions assessed in Experiment 2, but are less tractable for laboratory investigation.
