# Supplementary figures and images for: Intrinsic Valuation of Information in Decision Making under Uncertainty
Source: PLoS Comput Biol. 2016 Jul 14;12(7):e1005020. doi: 10.1371/journal.pcbi.1005020 (PMC4944922; doi:10.1371/journal.pcbi.1005020)

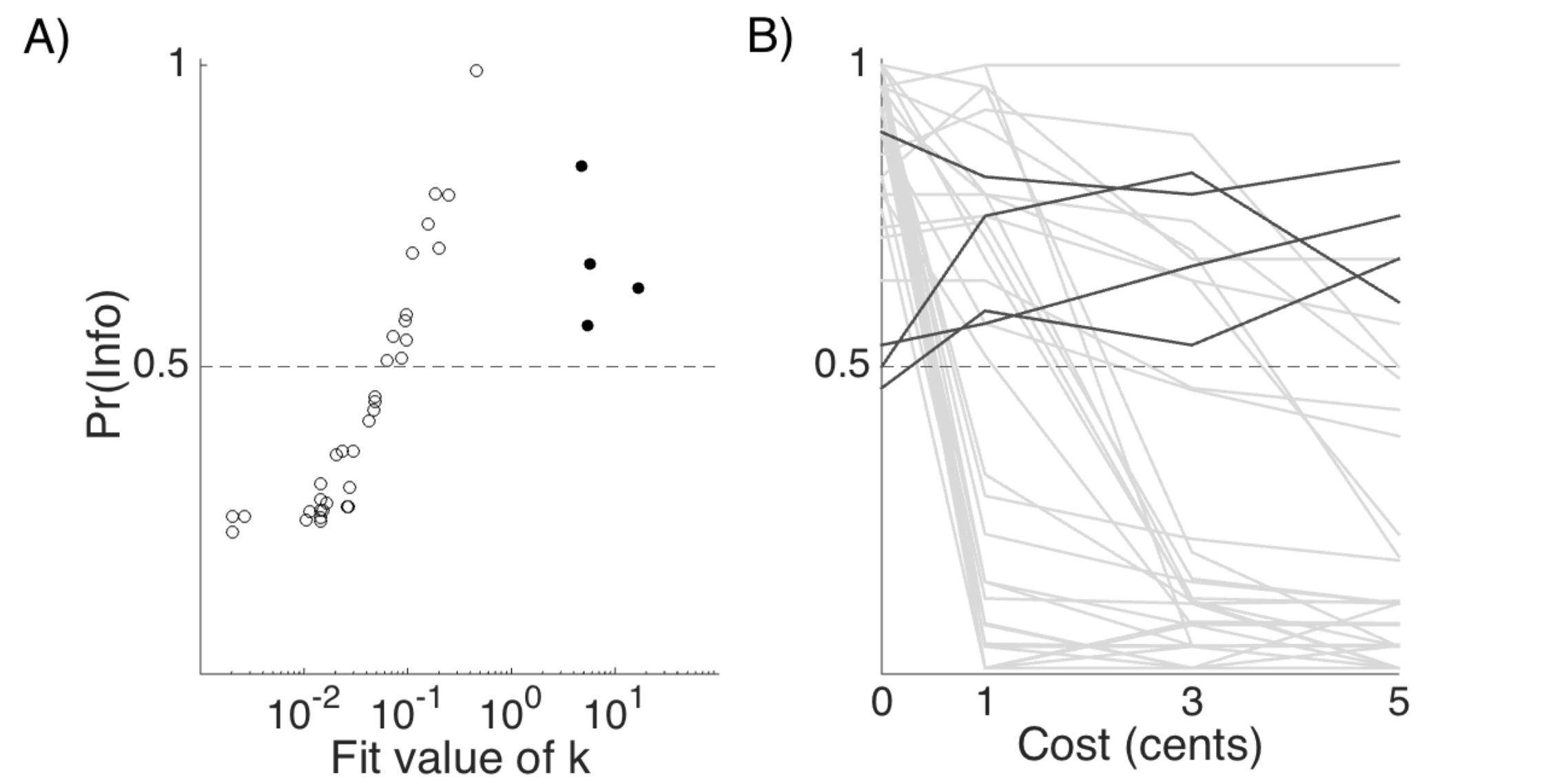

Supplement: S1 Fig — (TIFF) [file pcbi.1005020.s004.tiff]

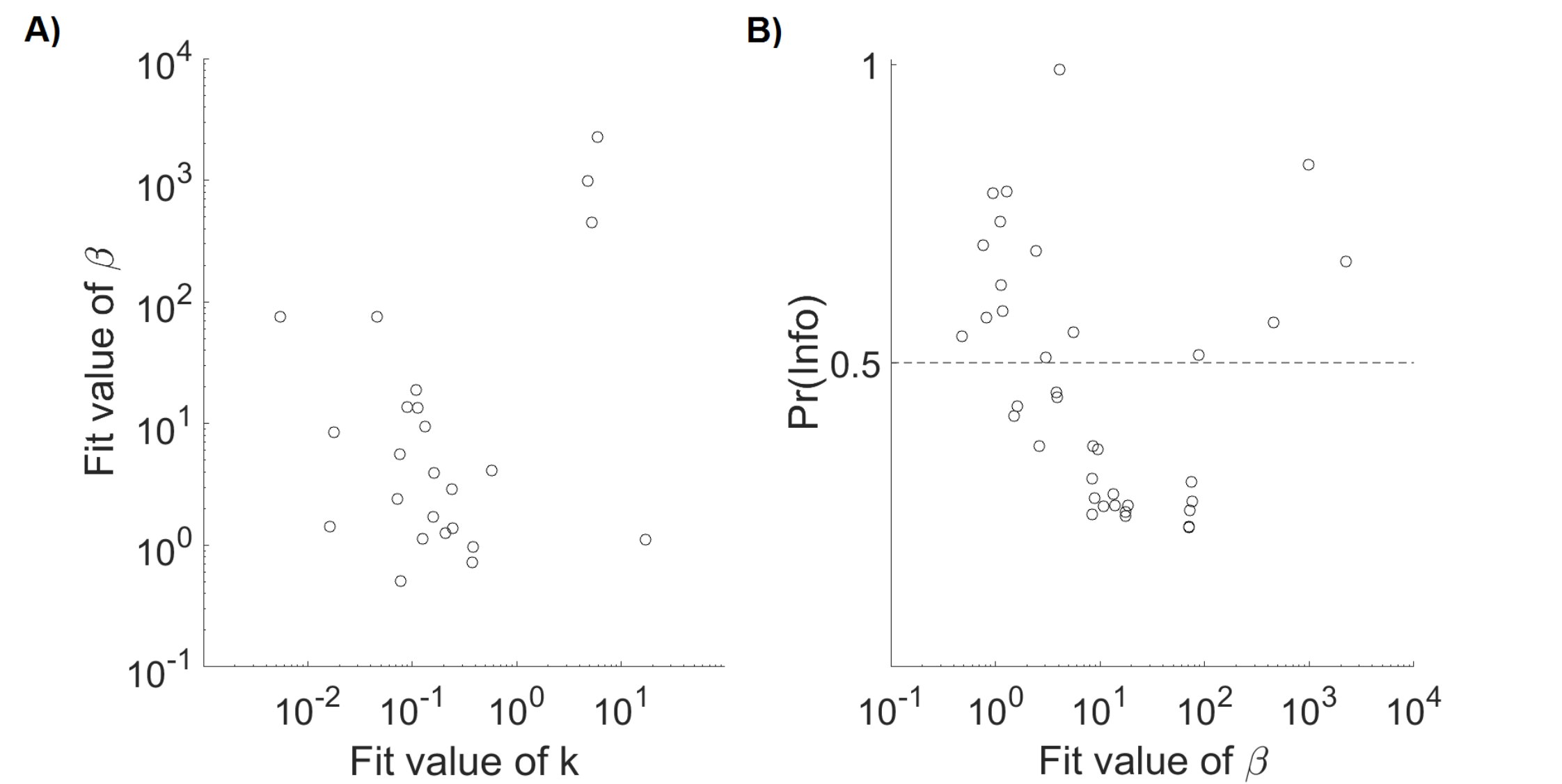

Supplement: S2 Fig — (TIFF) [file pcbi.1005020.s005.tiff]
